# The Relationship between the Infant Gut Microbiota and Allergy. The Role of *Bifidobacterium breve* and Prebiotic Oligosaccharides in the Activation of Anti-Allergic Mechanisms in Early Life

**DOI:** 10.3390/nu12040946

**Published:** 2020-03-29

**Authors:** Bożena Cukrowska, Joanna B. Bierła, Magdalena Zakrzewska, Mark Klukowski, Elżbieta Maciorkowska

**Affiliations:** 1 Department of Pathology, The Children Memorial Health Institute, Aleja Dzieci Polskich 20, 04-730 Warsaw, Poland; j.bierla@ipczd.pl; 2 Department of Developmental Age Medicine and Paediatric Nursing, Faculty of Health Sciences, Medical University of Bialystok, Szpitalna St. 37, 15-295 Białystok, Poland; magdalena.maciorkowska@com.pl (M.Z.); emaciorkowska@o2.pl (E.M.); 3 Department of Pediatrics and Pulmonary Diseases, Faculty of Health Sciences, Medical University of Bialystok, Jerzego Waszyngtona St. 17, 15-274 Białystok, Poland; mark.klukowski@gmail.com

**Keywords:** microbiota, gut, allergy, prevention, synbiotics, *Bifidobacterium breve*

## Abstract

The increase in allergy prevalence observed in recent decades may be a consequence of early intestinal dysbiosis. The intestinal microbiota is formed in the first 1000 days of life, when it is particularly sensitive to various factors, such as the composition of the mother’s microbiota, type of delivery, infant’s diet, number of siblings, contact with animals, and antibiotic therapy. Breastfeeding and vaginal birth favorably affect the formation of an infant’s intestinal microbiota and protect against allergy development. The intestinal microbiota of these infants is characterized by an early dominance of *Bifidobacterium*, which may have a significant impact on the development of immune tolerance. *Bifidobacterium breve* is a species commonly isolated from the intestines of healthy breastfed infants and from human milk. This review outlines the most important environmental factors affecting microbiota formation and the importance of *Bifidobacterium* species (with a particular emphasis on *Bifidobacterium breve*) in microbiota modulation towards anti-allergic processes. In addition, we present the concept, which assumes that infant formulas containing specific probiotic *Bifidobacterium breve* strains and prebiotic oligosaccharides may be useful in allergy management in non-breastfed infants.

## 1. Introduction

Allergic diseases are one of the leading medical challenges in highly developed countries, where the proportion of affected individuals may already exceed 30% and is constantly rising [1,2]. The rise in allergy prevalence may be associated with the so-called ‘western lifestyle’: improved hygiene, frequent use of antibiotics, reduced family size, altered eating habits (consumption of highly processed foods), general urbanization, and limited contact with nature [3,4]. Not without significance is also the growing number of Caesarean sections (C-sections), which correlates with an increased incidence of food allergy (FA) and asthma [5,6]. All these factors, which are associated with progressive development of societies and increase the risk of allergies, also greatly affect the intestinal microbiota—currently considered to be a ‘super organ’, necessary for the proper functioning of the immune system and the development of immune tolerance [7]. The microbiota hypothesis of allergy development assumes that the microbiota programs the baby’s immature immune system, which affects their health in later years, and the increase in allergy prevalence may be a consequence of intestinal dysbiosis during the first months of life [8]. In this review, we discuss the most important environmental factors affecting early microbiota composition and the importance of bifidobacteria, with a particular emphasis on *Bifidobacterium breve* strains, in immune-system modulation towards anti-allergic processes. We also present the concept which assumes that infant formulas containing a combination of specific probiotic *Bifidobacterium breve* strains and prebiotic oligosaccharides may be useful in allergy management in non-breastfed infants.

## 2. Intestinal Microbiota Formation and Dysbiosis-Inducing Factors 

It is currently accepted that the intestinal microbiota begins developing right after birth, but there are studies suggesting that microbiota formation may start in the pre-natal period [9,10]. During vaginal delivery, the mother’s microbiota is the main source of microorganisms colonizing newborns [11,12]. *Bifidobacterium* spp. appear in breastfed newborns already on the 2nd day of life, and by the second week, they become the predominant bacterial genus in the gastrointestinal tract. The predominance of *Bifidobacterium* spp. persists until solid foods are introduced into the diet and the baby is weaned off breast milk [13]. Expanding the diet causes an increase in the number of bacteria from the *Bacteroidetes* and *Firmicutes* phyla. By the age of approximately 2–3 years, the baby’s microbiota is stabilized, and its composition resembles that of adults’ microbiota, with predominance of *Bacteroidetes* [14,15]. This natural development of gut microbiota is often disturbed by C-sections, formula feeding, and antibiotic therapy [16,17,18]—i.e., dysbiosis-inducing factors that correlate with an increased risk of allergies.

### 2.1. Cesarean Sections, the Microbiota, and Allergy Development

Newborns born by C-section are deprived of exposure to the mother’s microbiota, which results in a dysbiosis observable already in the first days of life [13]. The intestinal microbiota of these neonates appears to be less diverse, in terms of bacterial species, than the microbiota of vaginally-delivered babies [19]. Moreover, unlike in vaginally-born newborns, bifidobacteria are still absent in C-section babies at the age of 3 days, despite breastfeeding [13]. Stools of C-section babies show reduced numbers of *Escherichia coli* and *Bacteroides*, with increased numbers of *Clostridium difficile* [20], and may harbor potentially pathogenic bacteria, such as *Klebsiella*, *Enterococcus*, and *Clostridium* [12,13,19].

The results of our study showed that C-section deliveries also influence the microbiota of babies born prematurely [21]. The intestinal microbiome of pre-term C-section babies, in comparison with vaginally-born pre-term newborns, showed a lack of *Bacteroides* in the first 8 days of life, and this status remained unchanged for the entire follow-up period of 8 weeks. Other researchers also point out that gut microbial dysbiosis associated with C-sections results in long-lasting disturbances of microbiota homeostasis [17]. Reduced biodiversity of microbiota, with a lower abundance of *Bacteroidetes* in C-section-delivered babies, was demonstrated to persist at least until the age of 2 years, i.e., until microbiota development has been completed [20]. Interestingly, analysis of peripheral blood cytokine profiles in these infants showed an imbalance in pro-inflammatory T helper type 1 (Th1) and pro-allergic T helper type 2 (Th2) cells. Infants born through C-section had significantly lower levels of the Th1-associated chemokines CXCL10 and CXCL11, which directed their immune response towards pro-allergic Th2-mediated reactions. 

Epidemiological studies have confirmed that Cesarean births correlate with an increased incidence of FA and asthma [5,6,22,23,24]. The latest American cohort study in children born in the state of New York (*n* = 1356) in the years 2008–2010 showed that emergency C-section delivery was associated with a higher relative risk (RR) of wheezing (RR = 2.47, 95% CI 1.31–4.66) and FA (RR = 3.02, 95% CI 1.26–7.25) [22]. A cohort study conducted by Wu et al. in 136,098 children showed that the risk of developing asthma in 4.5–6-year-old children with multiple risk factors, including C-section, increased more than sevenfold [25]. The odd ratio (OR) was 7.77 (95% CI 6.25–9.65), when, in addition to cesarean delivery, there were also other factors adversely affecting the intestinal microbiota, such as maternal and infant exposure to antibiotics, as well as a lack of siblings at home [26].

### 2.2. Antibiotics, Microbiota, and Allergy Development

Exposure to antibiotics is another important factor that shapes the intestinal microbiota. In the short term, antibiotic-induced changes in the intestinal microbiota may contribute to the pathogenesis of necrotizing enterocolitis (NEC) and antibiotic-associated diarrhea [27,28]. In the long term, antibiotic therapy in infants and young children may have a significant impact on the process of microbiota development and on microbial programming of anti-allergic mechanisms later in life [29,30]. Table 1 presents a summary of studies showing the effect of antibiotic exposure in perinatal and early post-natal periods on the composition of intestinal microbiota [26,31,32,33,34,35,36,37,38]. Studies in children from the age of 0 to 3 years showed that the microbiota of antibiotic-treated children was less diverse, with a lower abundance of the phylum *Actinobacteria* (with the genus *Bifidobacterium),* the genus *Lactobacillus, Bacteroides*, and had fewer stable bacterial communities in comparison with that in untreated children [38].

The impact of antibiotic therapy on the risk of allergy development was very thoroughly discussed in a 2018 review by Obiakor et al. [28]. The authors emphasized that an increase in the use of antibiotics in the early period of development observed in highly developed societies correlates with an increase in allergy rates. A meta-analysis published in 2018 including 34 studies and 340,428 patients showed that exposure to antibiotics during the first 2 years of life was associated with an increased risk of hay fever, eczema, and FA later in life [39]. Other studies not evaluated in this meta-analysis presented in Table 2 showed that antibiotic therapy in early life is also associated with a higher risk of wheezing and asthma [40,41,42,43,44,45,46,47].

### 2.3. Infant Diet, Microbiota, and Allergy Development

Breast milk contains vast amounts of biologically active components that have a significant impact on the development of the gut microbiota [48]. Exclusively breastfed infants show a predominance of *Bifidobacterium* species in their intestines [12,14]. The microbiota of formula-fed infants is far more diverse, with a predominance of staphylococci, *Bacteroides*, clostridia, enterococci, enterobacteria, and generally lower numbers of *Bifidobacterium* and *Lactobacillus* species [12,15]. *Bifidobacterium* species can multiply in the intestines of breastfed infants due to the presence of unique human milk oligosaccharides (HMOs) [49]. HMOs are natural prebiotics that are resistant to digestive enzymes and when unchanged reach the large intestine, where they are a selective substrate for bifidobacteria [50]. Although HMOs are considered to be ’bifidogenic’, it should be noted that only certain *Bifidobacterium* species, especially those present in the infant intestine, i.e., *Bifidobacterium breve*, *Bifidobacterium longum* subspecies *infantis,* and *Bifidobacterium bifidum*, have a high capacity for utilizing HMOs. 

#### 2.3.1. Human Breast Milk—A Natural Synbiotic

HMOs are not the only bioactive components that influence the formation of the intestinal microbiota [51]. Recent studies revealed that breast milk is not sterile and contains live probiotic *Lactobacillus* and *Bifidobacterium* species [52]. Apart from these two genera, *Streptococcus* and *Staphylococcus* are also present in human milk. The most commonly isolated *Bifidobacterium* species is *Bifidobacterium breve* and those of the genus *Lactobacillus* are *Lactobacillus salivarius* and *Lactobacillus fermentum* [52,53]. 

It can therefore be stated that breast milk is a natural synbiotic, containing both probiotics and prebiotics [51]. The composition of breast milk microbiota, like that of the intestinal microbiota, depends on many factors: the composition of the mother’s intestinal and skin microbiota, the state of her health, and exposure to medications, particularly antibiotics [54,55,56,57,58,59,60,61] (Table 3).

Antibiotic therapy during pregnancy and lactation drastically reduces *Lactobacillus* and *Bifidobacterium* counts in milk [54]. Studies have shown the complete absence of bifidobacteria in the breast milk of more than 50% of women who received antibiotics during pregnancy or lactation [54]. A significant decrease in diversity and the number of *Bifidobacterium* and *Lactobacillus* bacteria was also observed in the breast milk of women who gave birth via C-section, compared to that of women who gave birth vaginally [55,56]. The breast milk of obese mothers was also shown to contain lower numbers of bifidobacteria and higher numbers of *Staphylococcus* spp. and to demonstrate an altered immunomodulatory capacity associated with a decrease in the concentration of certain immunoregulatory proteins (e.g., growth transforming factor *β*2 [TGF-*β*2] and soluble CD14 molecules) [57]. 

#### 2.3.2. Human Milk and Allergy Prevention

Despite the observed differences in the composition of breast milk, studies confirmed that breastfeeding protects the baby against the development of allergies. Exclusive breastfeeding for at least 3 months was shown to reduce the risk of developing atopic dermatitis (AD), even in genetically predisposed children [60]. An observational study in 3296 children (Canadian Healthy Infant Longitudinal Development Birth Cohort) showed that, in comparison to exclusive breastfeeding for the first 3 months of life, a different diet, such as formula-based feeding (OR = 2.14, 95% CI 1.37–3.35) or breastfeeding with formula supplementation (OR = 1.73, 95% CI 1.17–2.57) increased the risk of developing asthma by the age of 3 years by about twofold [61]. 

Chu et al. attempted to answer the question of whether exclusive breastfeeding may decrease the risk of allergy in children born by C-section [62]. Their study confirmed that C-sections without medical indication were significantly associated with elevated risk of asthma at 6 years of age (OR = 1.58, 95% CI 1.17–2.13). However, this risk was attenuated in children who were breastfed exclusively for the first six months of life (OR = 1.39, 95% CI 0.92–2.10). By contrast, the risk was more prominent in children with non-exclusive breastfeeding or formula feeding (OR = 1.91, 95% CI 1.22–2.99).

## 3. Early Life Dysbiosis and Allergic Diseases

**Figure 1:**
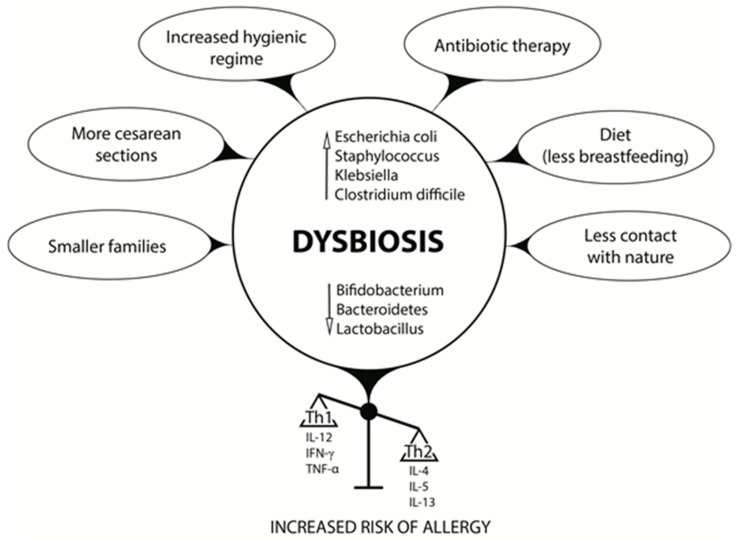
Factors affecting the formation of intestinal microbiota that induce dysbiosis and increase the risk of allergy.

The first 1000 days of life, which encompass both the pre- and post-natal periods of gut ecosystem formation, are the period of intestinal microbiota development. The intestinal microbiota is a new organ, which programs the immune system and affects the metabolism and other organs via the so-called “gut microbiota–organ axes” (e.g., the gut–brain axis, gut–skin axis, gut–lung axis, gut–liver axis) [9]. The intestinal microbiota of healthy, term, vaginally-born, breastfed infants seems to shape the immature immunity of newborns towards developing an immune tolerance. These microbiota-associated phenomena (immune system programming and regulatory effects on organ function) were described in a 2018 review paper by Cukrowska [8]). Thus, early intestinal dysbiosis induced by various factors (C-section, formula feeding, antibiotic therapy, smaller families, less contact with nature) may negatively influence the development of immune tolerance by disrupting the mechanisms regulating the balance between Th1 and Th2 cells and finally may activate pro-allergic processes and increased risk of allergy (Figure 1). Allergies are chronic diseases in which the Th2 arm of the adaptive immune response is dominated. The microbiota has a crucial role in generating a balanced immune phenotype that involves maturation of the Th1 cells response and the development of T regulatory (Treg) cells, which suppress the Th2 phenotype [63]. 

Comparisons of the intestinal microbiota of children with allergies with that of healthy children show that children with allergic diseases primarily have a reduced diversity of their gut microbiota and low abundance of *Bifidobacterium*, *Lactobacillus,* and *Bacteroides* [64,65,66,67,68,69,70,71,72,73,74]. Prospective studies suggest that intestinal dysbiosis in early life precedes the development of allergy in older children. Kalliomaki et al. showed that the decrease in the number of *Bifidobacterium* species and the increase in the number of *Clostridium* species (a microbiotic profile similar to that in babies born by C-section) observed in 3-week-old newborns was associated with the development of atopy (confirmed by skin-prick tests) within the first year of life [65]. Next-generation sequencing methods and metagenomic approaches showed that infants with immunoglobulin (Ig)E-associated AD have a lower microbiota diversity and a lower diversity of the phylum *Bacteroidetes* at one month of life, compared with a control group of up to 2-year-old infants without allergic manifestations [66]. Another study demonstrated that decreased bacterial diversity in the 1st year of life was associated with an increased risk of allergic diseases at 6 years of age [67] and an increased risk of asthma at 7 years of age [68]. Furthermore, lowering *Bifidobacterium* and *Lactobacillus* counts at 1–2 months of age increased the odds of developing an allergy by the age of 5 years [69].

The recently published study by Fieten et al. (2018) aimed to identify the fecal microbial signatures of FA in children with AD [70]. The authors identified six bacterial species which may determine the presence or absence of FA. These included three *Bifidobacterium* species: *Bifidobacterium breve*, *Bifidobacterium pseudocatenulatum*, and *Bifidobacterium adolescents*. The other three species were: *Escherichia coli*, *Faecalibacterium prausnitzii*, and *Akkermansia muciniphila*. Interestingly, children with FA were observed to have decreased numbers of *Bifidobacterium breve,* i.e., the species which is typically found in the early infant microbiota and human breast milk.

## 4. Intestinal Microbiota, Immunity, and the Development of Immune Tolerance

During fetal life, the immune system develops towards a pro-allergic Th2 cytokine profile, and its ability to produce Th1 cytokines (e.g., interleukin (IL)-12, interferon (IFN)-gamma) is impaired [71]. In addition, the immune system of newborns is immature, neonatal lymphocytes are referred to as naïve, i.e., never before exposed to external antigens, and the functioning of the intestinal barrier is disturbed due to the lack of secretory IgA, mucus, and microbiota, i.e., the outermost layers of the intestinal surface, which protect against pathogens, allergens, and toxins [72]. Thus, in early life, the baby’s immune system is more susceptible to the development of allergic reactions. Gut-colonizing bacteria are among the first antigens that activate the defense mechanisms, help to seal the intestinal epithelial barrier, establish immune tolerance, and modify the body’s response to potential allergens [73]. Our recent transmission electron microscopy study demonstrated that the gut microbiota improves the immature intestinal epithelium in germ-free (GF) mice [74]. The study showed the brush border of GF-mouse enterocytes to be arranged irregularly, with decreased numbers of cytoskeletal microfilaments and a lack of elongation into the terminal web. *Lactobacillus* species, obtained from the stools of healthy infants, significantly improved brush-border architecture and induced an increase in the expression of intraepithelial junction proteins (zonulin, occludin). Other experimental studies showed that colonization of GF piglets or mice with components of the gut microbiota found in healthy infants’ stools, such as non-pathogenic *Escherichia coli*, *Bifidobacterium longum,* or *Lactobacillus* strains, induced production of secretory IgA and activated CD4+Fox3+ Treg cells [75,76,77,78]. The mechanism of Treg action is associated with the release of cytokines (IL-10 and TGF-*β*1) responsible for maintaining homeostasis between Th1 and Th2 cells [79]. Treg activation is one of the basic mechanisms of immune tolerance induction. Disturbed functioning of these cells results in a Th1/Th2 imbalance and produces an abnormal immunological response to external allergens [80].

## 5. The Role of *Bifidobacterium breve* in Anti-Allergic Mechanism Activation—The Importance of Strain Selection

The genus *Bifidobacterium* belongs to the phylum *Actinobacteria* and comprises over 45 species, including *Bifidobacterium breve*, i.e., the bacterium most commonly isolated from healthy intestines of breastfed infants [12]. This species is also most commonly isolated from human breast milk [54]. In fact, human breast milk may be the main source of *Bifidobacterium breve* strains that colonize a newborn’s intestines shortly after birth [12]. *Bifidobacterium breve*, which is the main constituent of the intestinal microbiota of healthy newborns, is responsible for the development of intestinal biocenosis as well as for the activation of the immature immune system [81].

The anti-allergic capability of *Bifidobacterium breve* species was demonstrated in a number of in vitro and experimental animal studies [82,83]. Experimental models of FA to ovalbumin (OVA) in mice showed that out of various strains of *Bifidobacterium breve*, *Bifidobacterium infantis*, *Bifidobacterium animalis*, *Lactobacillus plantarum*, and *Lactobacillus rhamnosus*, it was *Bifidobacterium breve* M-16V that was the most effective in activating an anti-allergic mechanism [83]. In contrast to other evaluated bifidobacteria, only *Bifidobacterium breve* M-16V (administered orally) significantly inhibited airway reactivity to methacholine and reduced acute allergic skin reactions to OVA. This strain also reduced the number of eosinophils in bronchoalveolar lavage fluid, decreased OVA-specific IgE and IgG1 levels in peripheral blood, and inhibited the production of pro-allergic cytokines, such as IL-4 and IL-5 in splenocyte cultures [83]. In vitro analyses demonstrated that although *Bifidobacterium breve* M-16V suppressed the production of OVA-induced total IgE and IL-4, it activated the secretion of IFN-gamma (but not IL-12) and IL-10 at the same time, affecting the maintenance of the systemic Th1/Th2 balance [82]. 

### 5.1. The Impact of Bifidobacterium breve M-16V on Infant Immunity

The potential beneficial effects of *Bifidobacterium breve* M-16V strain on infant health (observed also in pre-term infants) have been recently presented in a review paper by Wong et al. [84].

Pre-clinical experimental studies and clinical trials showed that *Bifidobacterium breve* M-16V may protect against the development of allergies through the impact on the intestinal microbiota, intestinal epithelial barrier, and immune system. This strain promotes bifidobacterial colonization during early infancy and stimulates secretory IgA production [85]. Clinical studies in premature infants showed that *Bifidobacterium breve* M-16V administration for four weeks leads to higher levels of short chain fatty acids (especially acetate) in infant stools. This phenomenon is often associated with an abundance of bifidobacteria, which may improve epithelial-cell barrier function [86]. Studies in an experimental rat model demonstrated a direct activation of immature immunity by *Bifidobacterium breve* M-16V [87]. Oral supplementation with this strain enhanced the process of naïve T-cell homing to mesenteric lymph nodes and the retention of activated T cells in the intraepithelial compartment, probably via increased expression of integrin αE*β*7. Administration of *Bifidobacterium breve* M-16V to pre-term newborns, initiated several hours after birth, induced a significant increase both in serum TGF-*β*1 levels and expression of TGF-*β* signaling molecule Smad3 [88]. These results suggest that *Bifidobacterium breve* M-16V is able to stimulate regulatory TGF-*β*1 produced by Treg cells even in premature newborns. The ability to activate Treg cells is of great importance in pre-term newborns, who are at high risk of developing NEC and late-onset sepsis. *Bifidobacterium breve* M-16V, colonizing the gut and inducing epithelial barrier maturation, may protect against pathogenic bacteria and their translocation; additionally, this strain is able to induce anti-inflammatory processes, e.g., by increasing IFN-gamma secretion [83]. However, the activation of pro-inflammatory cytokine profile can be actively regulated by suppressive cytokines produced by Treg cells, such as TGF-*β*1 or IL-10 [79]. Another mechanism for the controlled activation of inflammatory processes in premature babies by *Bifidobacterium breve* may be associated with the modulation of the expression of toll-like receptors (TLRs) located in the gut epithelium. Oral administration of *Bifidobacterium breve* M-16V to the experimental NEC rats significantly decreased the expression of TLR-4, enhanced the expression of TLR-2, and suppressed pro-inflammatory cytokines, including IL-1 beta, IL-6 and tumor necrosis factor alpha that resulted from NEC induction [89]. It is known that TLRs are the main receptors for interaction between the gut epithelium and the microbiota that enables development of both the intestinal epithelial barrier and the immune homeostasis [90]. 

### 5.2. Bifidobacterium breve M-16V and Allergic Diseases 

Several interventional studies suggest that *Bifidobacterium breve* M-16V may prevent or reduce the severity of allergic conditions, including FA, AD, allergic rhinitis, and asthma [91,92,93]. The first relevant randomized controlled trial was performed in 2003 and involved 15 bifidobacteria-deficient infants with AD [91]. The authors reported that *Bifidobacterium breve* M-16V administration was not only effective in improving the severity of allergic symptoms but also significantly increased the number of *Bifidobacterium* species and decreased the number of total aerobes in the gut microbiota.

The effectiveness of *Bifidobacterium breve* M-16V in allergy management was also demonstrated when this strain was used as a mixture in combination with *Bifidobacterium longum* BB536 and *Bifidobacterium infantis* M-63, i.e., other species that can also be found in breastfed infants [92]. The mixture was administered orally for four weeks in children with seasonal allergic rhinitis as part of a randomized double-blind placebo-controlled trial. Del Giudice et al. showed that administration of this mixture reduced the symptoms of pollen-induced IgE-mediated allergic rhinitis and intermittent asthma and improved the quality of life. By contrast, allergic symptoms and the quality of life worsened in the placebo group [92]. Enomoto et al. reported that pre-natal *Bifidobacterium breve* M-16V and *Bifidobacterium longum* BB536 supplementation in pregnant women and a subsequent post-natal supplementation in newborns may activate anti-allergic mechanisms of the immature immune system and lower the risk of allergy development in infants [93]. This open trial involved administering a mixture of these two strains to 130 pregnant women, starting one month before delivery, and post-natally to their infants for six months. The control group included mother–infant pairs who did not receive probiotics. The risk of developing AD during the first 18 months of life was significantly reduced in the infants from the probiotic group (OR = 0.231 (95% CI 0.084–0.628) and 0.304 (0.105–0.892) at 10 and 18 months of age, respectively).

## 6. Synbiotic Formulas Fortified with *Bifidobacterium breve*—The Effects on the Intestinal Microbiota and the Role in Allergy Management

The concept of synbiotics—based on the synergistic actions of probiotics and prebiotics resulting in an optimal composition of the infant gut microbiota—seems to be useful in allergy management in non-breastfed infants. Synbiotic formulas are defined as infant formulas fortified with two ingredients found in human breast milk: prebiotic oligosaccharides and probiotic bacteria [94,95]. Prebiotics are oligosaccharides that provide sustenance for bacteria. Preparations containing galactooligosaccharides (GOS), fructooligosaccharides (FOS), 2′-fukosyllactose, and/or lacto-N-neo-tetraose are examples of commonly used and studied products for infant formula supplementation. The probiotics added to synbiotic formulas are most commonly bacteria (isolated from breast milk and/or healthy infant stools) characterized by experimentally proven immunomodulatory effects. 

Molecular analyses have demonstrated that infant-associated *Bifidobacterium breve* strains express high abundance of genes that are predicted to encode carbohydrate-utilizing enzymes that catabolize sucrose, fructose, lactose, glucose, and galactose [96]. Interestingly, bifidobacteria, including *Bifidobacterium breve* species, express also genes involved in maltodextrin utilization [96]. Thus, it seems that not only HMOs, lactose or other known prebiotic oligosaccharides can serve as prebiotic substrates for specific *Bifidobacterium* strains, but also maltodextrin present in formulas intended for premature infants may be utilized by *Bifidobacterium breve*. However, there are currently no studies that would support this hypothesis, and it should be emphasized that experimental research carried out on a pre-term pig model of spontaneous NEC suggests that the presence of maltodextrin in formula, unlike lactose, may increase the risk of NEC [97]. 

Chua et al. studied the effects of synbiotic infant formulas on C-section-associated dysbiosis [98]. The study compared the effects of formulas enriched with scGOS/lcFOS and *Bifidobacterium breve* M-16V, with scGOS/lcFOS alone, and not enriched with any additional bioactive components. The control group consisted of newborns with an optimal microbiota composition due to their vaginal birth and subsequent breastfeeding. Out of all the evaluated formulas, the synbiotic formula showed the most pronounced ameliorating effect on C-section-associated dysbiosis. Newborns fed with synbiotic formula were observed to have an increase in bifidobacteria numbers that started on the 3rd day of life and was sustained for 16 weeks. The amount of bifidobacteria in the experimental group was comparable to that in vaginally-born, breastfed infants during the entire study period. This bifidogenic effect of synbiotic formulas was also confirmed in young children, aged 1–3 years, in whom a 3-month administration of a synbiotic formula resulted in an increase in the number of *Bifidobacterium* species [99]. 

These results show that synbiotic formulas may have a beneficial long-term effect (similar to that of breast milk) on the composition of the intestinal microbiota in infants at risk for early dysbiosis, i.e., in newborns born by C-section or children pre-/post-natally exposed to antibiotic therapy. In addition, experimental in vivo studies on a mouse model of allergy showed that administration of a synbiotic consisting of *Bifidobacterium breve* M-16V and GOS/FOS or FOS mixture could protect against the development of cow’s milk allergy [100]. Administration of *Bifidobacterium breve* M-16V in combination with specific β-lactoglobulin-derived peptides and prebiotic scFOS/lcFOS in mice was shown to prevent the acute allergic skin response and clinical signs of allergy in mice challenged intradermally with whole whey protein. Cytokine analyses conducted in cultures of lymphocytes isolated from the spleen, mesenteric lymph nodes, and the intestinal lamina propria demonstrated that intestinal Th1/Th2 balance was a hallmark of the mechanism underlying the protective effect of the administered mixture [100]. The other pre-clinical study revealed that administration of *Bifidobacterium breve* M-16V in combination with non-digestible oligosaccharides could suppress pulmonary airway inflammation in murine OVA-induced asthma models [101]. Synbiotic administration induced a Treg response in the airways by increasing IL-10 and Foxp3 transcription and reducing airway re-modeling. Thus, the presented experimental studies show that dietary supplementation with synbiotics may be effective in prevention of allergy development.

The question remains whether synbiotic formulas containing probiotic strains and prebiotic oligosaccharides may be of importance in immunological and nutritional programming in infants at a higher risk of allergy, on one hand caused by genetic predisposition and on the other by early dysbiosis resulting from C-section or antibiotic treatment. 

## 7. Conclusions 

Human milk, which contains prebiotic oligosaccharides and probiotic bacteria, is a natural synbiotic, with known beneficial effects on the intestinal microbiota, intestinal barrier, and immune system. Breastfeeding is the optimal source of nutrition and protects against the development of allergies, even in children with a genetic predisposition and C-section- or antibiotic-therapy-induced intestinal dysbiosis. Synbiotic formulas are meant to reflect the composition of human breast milk and protect non-breastfed infants against intestinal dysbiosis and its long-term consequences, such as the development of allergic conditions.

## Figures and Tables

**Table 1:** Summary of studies demonstrating the effects of antibiotic treatment in the pre-natal period and early post-natal life on the gut microbiota.

Age of Exposure to Antibiotics	Age at Evaluation	Study Description	Type of Antibiotics	Effects on Microbiota	References
Prenatal (maternal intrapartum antibiotic prophylaxis for Group B *Streptococcus* or C-sections)	3, 12 months after birth	Term infants (*n* = 198)	Cefazolin, penicillin	↓the genus *Bacteroides* and *Parabacteroides*; ↑*Enterococcus* and *Clostridium;* differences persisted 1 year after birth in infants with emergency C-sections	Azad et al. (2016) [30]
Prenatal (maternal intrapartum antibiotic prophylaxis for Group B *Streptococcus)*	10, 30, 90 days after birth	Term infants (*n* = 40)	Penicillin	↓the phylum *Actinobacteria* and *Bacteroidetes* after 10 days; ↑the phylum *Firmicutes* after 10 and 90 days	Nogacka et al. (2017) [31]
The first 2 days of life	4, 8 weeks after finishing antibiotic treatment	Term infants (*n* = 18)	Ampicillin, gentamycin	↑the phylum *Proteobacteria* ↓the phylum *Actinobacteria* ↓the genus *Bifidobacterium* and *Lactobacillus* after 4 weeks ↑the phylum *Proteobacteria* ↑the family *Enterobacteriaceae* and ↑the genus *Clostridium* after 8 weeks	Fouthy et al. (2012) [32]
The first 4 days of life	5 days, 1, 2 months after birth	Term infants (*n* = 26)	Broad–spectrum antibiotics	↓diversity of *Bifidobacterium* and ↑*Enterococcus* after 5 days ↑*Enterobacteriaceae* after 1 and 2 months	Tanaka et al. (2009) [33]
The first 7 days of life	7 days, 1, 3 months after birth	Term infants vaginally born and breastfed (*n* = 45)	Penicillin, amoxicillin/clavulamic acid, gentamycin, cefdazidine	↓the phylum *Bacteroidetes* at all time points A delay in *Bacteroidetes* colonization persisted for 3 months	Eck et al. (2020) [34]
The first 7 days of life	1, 2, 3 weeks after birth	Preterm infants ≤32 weeks gestational age (*n* = 74)	Ampicillin and gentamycin	↓diversity and ↑*Enterobacter* after 2 and 3 weeks	Greenwood et al. (2014) [35]
Prenatal (maternal intrapartum antibiotic prophylaxis for Group B *Streptococcus*) and/or the first 14 days of life	10, 30, 90 days after birth	Very low birth weight pre-term infants	Penicillin, ampicillin, ampicillin with erythromycin	↑the phylum *Firmicutes* and *Proteobacteria* ↓the phylum *Actinobacteria* after 30 days	Arboleya et al. (2015) [36] Arboleya et al. (2016) [37]
The first 3 years of life	Monthly collection of samples	Term infants (*n* = 39) at 2 months of age observed for 3 years (2–36 months of life)	Different antibiotics (9–15 antibiotic courses in the first 3 years of life)	↓diversity at the level of species and strain ↓*Bacteroides* Less stable community and ↑antibiotic resistance strains	Yassour et al. (2016) [38]

↓—a decrease in bacteria counts or diversity; ↑—an increase in bacteria counts or antibiotic resistance.

**Table 2:** Summary of studies demonstrating the impact of antibiotic exposure in the pre-natal period and early post-natal life on the risk of wheeze and asthma.

Age of Exposure to Antibiotics	Age at Evaluation	Type of Study	Type of Antibiotics	Impact on the Risk of Allergy	References
Prenatal (during pregnancy)	3 years	A retrospective cohort study	Different antibiotics	↑asthma, but only in children with familial risk	Lapin et al. (2015) [40]
Prenatal (during pregnancy)	Up to 5 years	A prospective birth cohort	Antibiotics for non-respiratory infections	↑asthma	Stensballe et al. (2013) [41]
Prenatal (during pregnancy) and the first year of life	3 and 6 years	A retrospective study	Different antibiotics	Postnatal exposure—↑asthma Prenatal exposure—↑asthma only until age3 years	Yoshida et al. (2018) [42]
Prenatal and the first year of life	2–10 years	A population- and register-based nested case-control study	Cephalosporins, sulphonamides, trimethoprim, macrolides, amoxicillin	↑asthma	Metsälä et al. (2015) [43]
Prenatal and the first year of life	Up to 7 years	A nationwide population based study with sibling analysis	Different antibiotics	↑asthma when exposed to antibiotics treating respiratory infections	Ortqvist et al. (2014) [44]
The first 6 months of life	2 years	A birth cohort study	Different antibiotics	↑wheezing, but not eczema and allergic sensitization	(Kummeling et al.2007) [45]
The first 6 months of life	12 years	A longitudinal cohort study	Different antibiotics	↑atopic asthma	Stromberg Celind et al. (2018) [46]
The first 3 years of life	15 years	A cohort study	Different antibiotics	↑asthma, but no asthma exacerbation	Ahmadizar et al. 2017 [47]

↓—reducing the risk of asthma or wheezing; ↑—increased the risk of asthma or wheezing.

**Table 3:** Maternal factors influencing the composition of breast milk microbiota.

Factor	Microbiota Change	References
Cesarean Section	↓*Bifidobacterium* ↓*Lactobacillus* ↓biodiversity ↑*Staphylococcus*	Khodayar-Pardo et al. (2014) [54] Cabrera-Rubio et al. (2016) [55]
Overweight and Obesity	↓*Bifidobacterium* ↓biodiversity ↑*Staphylococcus*	Collado et al. (2012) [56]
Antibiotic Therapy	↓*Bifidobacterium* ↓*Lactobacillus*	Soto et al. (2014) [57]
Allergy	↓*Bifidobacterium*	Grönlund et al. (2007) [58]
Celiac Disease	↓*Bifidobacterium*	Olivares et al. (2015) [59]

↓—a decrease in bacteria counts, ↑—an increase in bacteria counts.
